# Tunable Stiffness and Damping Study on Flexible PVC Cantilever Structure Embedded with MR Fluid

**DOI:** 10.3390/ma14175024

**Published:** 2021-09-02

**Authors:** Gunasekaran Ramkumar, Arul Jesu Gnanaprakasam, Marimuthu Thirumarimurugan

**Affiliations:** 1 Department of Mechanical Engineering, Coimbatore Institute of Technology, Coimbatore 641014, India; 2 Department of Chemical Engineering, Coimbatore Institute of Technology, Coimbatore 641014, India; gnanaprakasam1989@gmail.com (A.J.G.); thirumarimurugan@cit.edu.in (M.T.)

**Keywords:** MR fluid, tunable stiffness, cantilever structure, damping

## Abstract

The stiffness and damping of a flexible smart cantilever structure controlled by a magnetic field is investigated in this research. The cantilever structure is fabricated by using flexible polyvinyl chloride as a host structure of rectangular cross-section embedded with magnetorheological (MR) fluid. The deflection of the cantilever structure at the free end is used to analyze the stiffness change of the cantilever structure. The stiffness of the specimen with MR fluid at magnetic flux density of 0.171 T is greater than that of the specimen without subjected to magnetic field. The strength of the applied magnetic field is directly related to the structure’s stiffness. Under the influence of a magnetic field, the MR fluid embedded inside the flexible PVC cantilever structure significantly dampens the vibrations of the structure.

## 1. Introduction

Vibration damping is important in a wide range of structures in civil engineering, aerospace, marine, transportation, robotics, and other fields [1,2]. Vibration damping can be accomplished using different types of methods. Among these methods, passive vibration damping is the most fundamental, requiring no external energy to effect a change in the mechanical system. The feedback unit and sensors are not included in passive vibration damping. Passive damping devices include springs and shock absorbers. In the adaptive vibration damping method, a material with adjustable behavior is used to adapt to changes in the environment of the system. This system’s response can be adjusted continuously to achieve the desired results. There are two methods for converting a passive system to an adaptive system. One is active control, which involves adding additional external energy to the system, and the other is semi-active control, which involves changing the mechanical features of the system [3]. In active vibration control, smart materials such as piezoelectric, magnetostrictive, electrostrictive, shape memory alloy, and electroactive polymers are used as core material of the structure to control the vibration.

**Figure 1:**
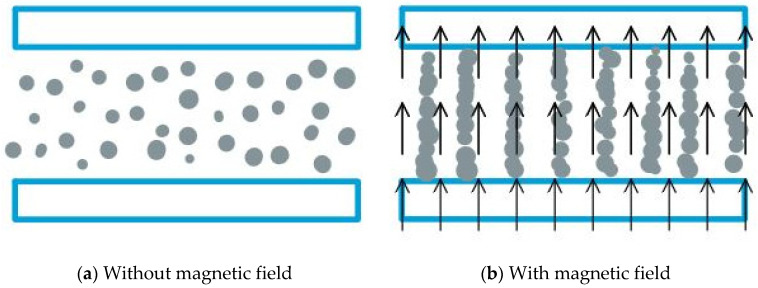
Working principle of MR fluid.

MR fluid are colloidal suspensions of magnetic particles which polarize and form particle chains under application of a magnetic field. This restricts particle movement, resulting in fluid motion restrictions. A fluid’s apparent viscosity varies in a reversible manner in reaction to a magnetic field. When MR fluids are exposed to a magnetic field, changes in elasticity, plasticity, and viscosity are observed. Due to the charge migration mechanism, a pseudo phase shift from liquid to solid occurs in the presence of a magnetic field, and it exhibits Bingham plastic fluid characteristics. By changing the applied electric/magnetic field, fluid motion resistance can be regulated. Electrorheological fluids require substantially higher voltage and current to operate than MR fluids. Shear stress is higher in MR fluid than in ER fluid [4]. MR fluids are less temperature sensitive and have a higher level of stability than ER fluids. The functioning concept of MR fluid is shown in Figure 1. When the external magnetic field is removed, the MR fluid returns to its original properties in milliseconds. Hence, MR fluid is used as a structural core component to alter the structure’s stiffness, damping, and modal characteristics [5,6,7]. The magnetic field controls MR fluid, which can be used as a core damping component inside the host structure [8,9,10]. Vibration damping with MR fluid can be passive, semi-active, or active. They are applied in clutches, brakes, suspension, throttle valves and flexible structures. In the areas of machines, devices, building, and structural elements, vibration suppression is one of the most challenging engineering challenges [11,12]. To improve the stiffness of such systems, MR fluid have been incorporated into the sandwich structures.

The use of MR fluids to examine sandwich structures is a recent scientific advancement. The vibration, dynamic, and stability characteristics of sandwich structures such as circular plates, shells, structures, panels, and cylindrical panels are investigated [13,14]. Many research works have concentrated on testing, numerical analysis, and mathematical modelling of field-dependent properties in the structure category [15,16,17]. Aluminum [18,19,20,21], polyethylene terephthalate [22], glass fiber reinforced polymer composite [23,24], and functionally graded carbon nanotube reinforced laminated composite [25] were used as sandwich structure base materials. The results from analysis of sandwich structures using MR fluids show that natural frequencies increase with increase in strength of magnetic field intensity. Vibration amplitude is minimized by application of magnetic field. Sandwich structures were also analyzed for rotating conditions and reinforcement of different composite layers. The stiffness change and damping characteristics of flexible polyvinyl chloride with MR fluid core are investigated in this work. The flexible polyvinyl chloride has excellent dimensional stability, abrasion resistance, and impact resistance. Medical tubing, artificial muscle, robotic grippers, automotive parts, building uses such as frames and roofs, electrical insulation, and other applications make flexible polyvinyl chloride a highly commercial material. Since the application of MR fluid in flexible polyvinyl chloride structures improves stiffness, adaptive structures for various applications can be devised.

## 2. Characterization of MR Fluid

**Figure 2:**
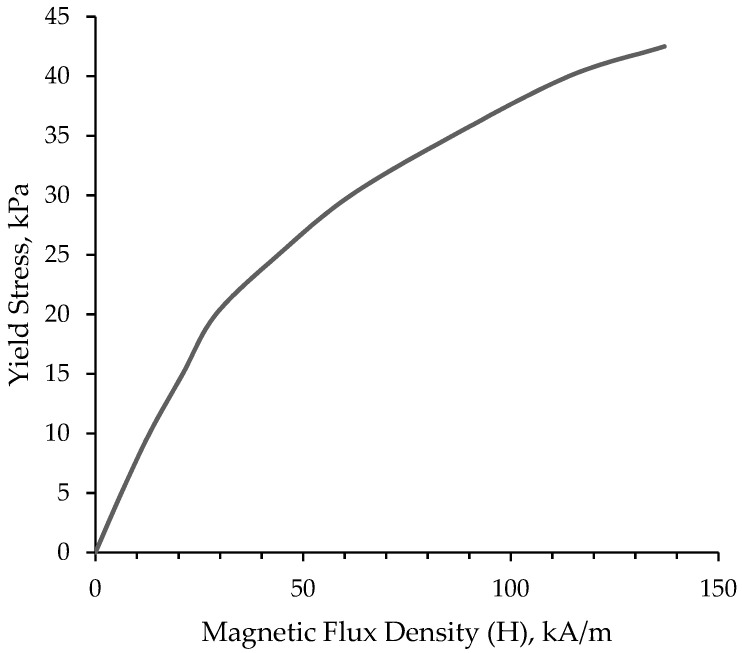
Characterization of MR fluid as yield stress vs Magnetic flux density plot (yield stress curve).

The behavior of MR fluid in presence of the magnetic field is studied before proceeding with sandwich structures embedded with MR fluid. The parallel plate rheometer is used to evaluate the change in rheological characteristics of fluid under shear loading conditions in the presence of an external magnetic field. When the magnetic field and flow directions are normal to each other, significant differences in fluid characteristics occur. Figure 2 depicts the typical response of MR fluid in shear, with yield stress displayed as a function of magnetic flux density. It shows that by increasing the magnetic flux density, the fluid viscosity increases (becomes semi-solid) as the magnetic particles orient in the direction of magnetic field. Thus, the resistance to the load applied is increased as the viscosity of the fluid is increased. Therefore, the stiffness of the structure is influenced by the MR fluid under magnetic field. Table 1 lists the properties of MR fluid.

## 3. Development of Flexible PVC Structure with MR Fluid

**Figure 3:**
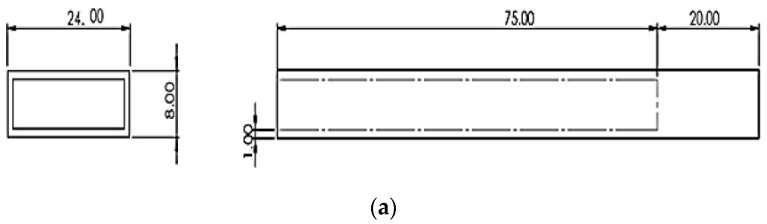

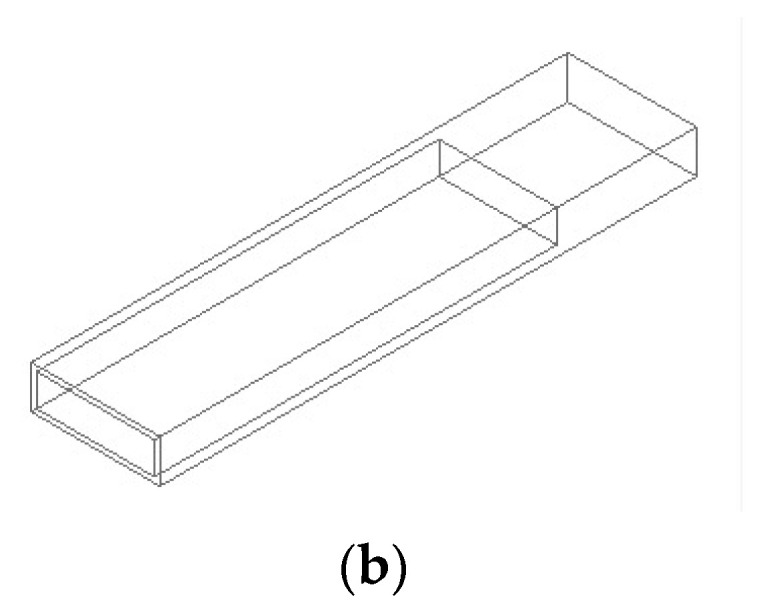
Schematic view of the flexible PVC–MR Fluid cantilever beam (**a**) Dimensional view of the specimen (all dimensions are in mm) (**b**) Wireframe model of the specimen.

The specimen consists of two materials. Flexible PVC is used as a host material and MR fluid is used as a core material of the cantilever beam. The extrusion process is used to produce a rectangular hollow cross section structure made of polyvinyl chloride. The polyvinyl chloride structure’s annular core portion is 75 mm long, while the clamped part is 20 mm long. After filling MR Fluid into the core using a syringe (Hindustan syringes, India) the end of the annular part is covered with PVC elastomer cap. Figure 3 shows the cantilever structure’s dimensions and the wireframe model.

## 4. Measurement of Magnetic Field Intensity

**Figure 4:**
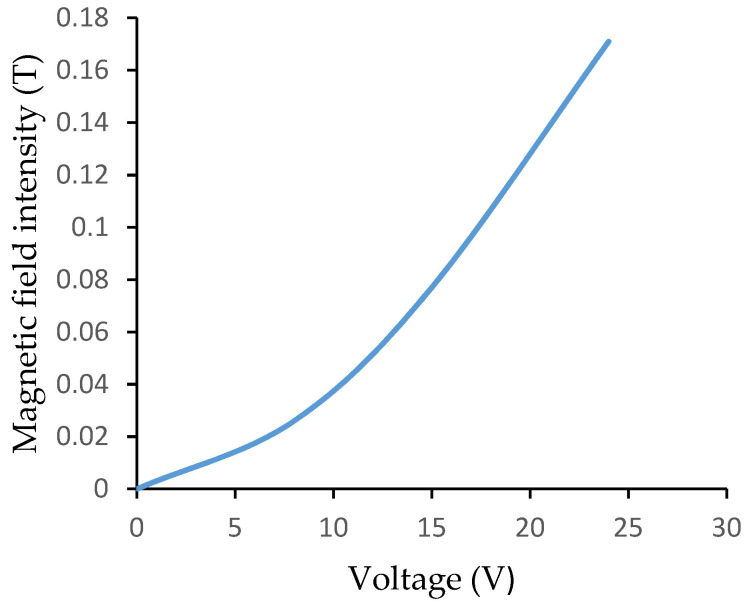
Excitation curve of electromagnet (Measurement of magnetic field intensity).

The electromagnet used in this research is studied for its magnetic field intensity as a function of applied voltage. The gaussmeter (Nunes Instruments, Coimbatore, India) is used to measure the magnetic strength at the distance of 4 mm from the electromagnet and the magnetic field intensity for different set of input voltages applied to the electromagnet is recorded. The measurement of magnetic field intensity is shown in Figure 4.

## 5. Experimentation

The vibration response and stiffness characterization tests are performed to investigate the damping characteristics and stiffness of the cantilever structure, respectively.

### 5.1. Vibration Response of the Cantilever Structure

**Figure 5:**
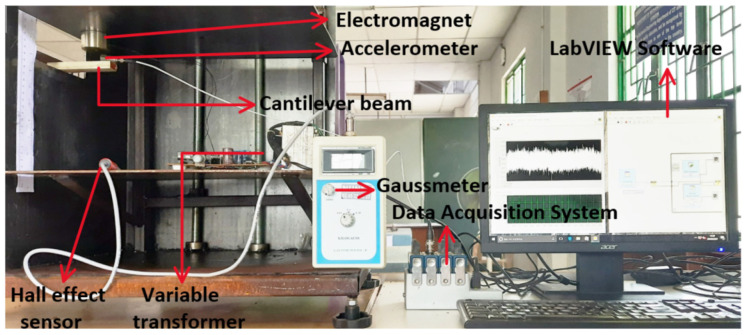
Experimental setup for vibration response of cantilever structure.

A sensor used to monitor vibration is a 3097 A1 piezoelectric accelerometer (PCB piezotronics, USA). It is positioned over the flexible PVC-MR fluid cantilever structure’s free end. As illustrated in Figure 5, the accelerometer is connected to a data acquisition device (DAQ NI 9234) to acquire signals from the sensor. Instrumentation is done using LabVIEW software (2012, National Instruments, India). Figure 4 shows a flexible PVC–MR fluid cantilever sandwich beam placed below the variable electromagnet. The variable electromagnet is 40 mm in diameter. The magnetic field is controlled by varying the voltage supplied using the transformer. The hall effect sensor of gaussmeter captures the magnetic flux density after the voltage supplied to electromagnet is kept constant. In LabVIEW software, programming is done to measure the vibration response of a cantilever structure using a time domain plot and spectral analysis. The time domain plot shows the amplitude in x-axis and time in y-axis. The power spectrum analysis plot shows the amplitude in x-axis and frequency in y-axis. The accelerometer signal is sent to DAQ for processing, and natural frequency and damping parameters are presented on the monitor.

The flexible PVC–MR fluid cantilever structure’s free vibration response without magnetic field (0 T) is determined by applying an initial disturbance to the structure and then removing the disturbance, resulting in free vibration in absence of magnetic field. The structure’s natural frequency and vibration response is captured in LabVIEW software. Similarly, the vibration response of the flexible PVC–MR fluid cantilever structure is determined with magnetic field at 0.171 T.

### 5.2. Stiffness Characterization of the Cantilever Structure

**Figure 6:**
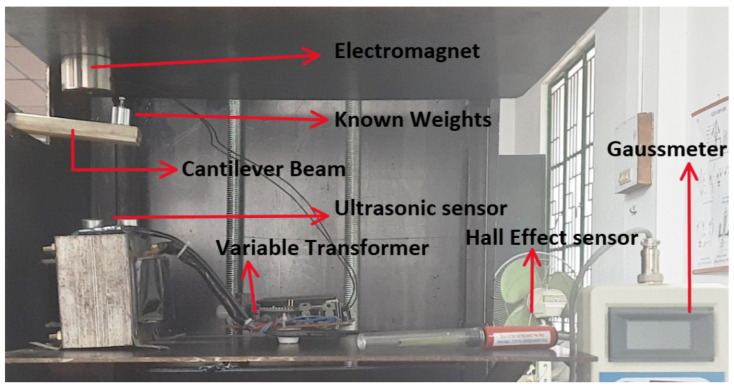
Experimental setup for load–displacement measurement.

The load–displacement relationship of the flexible PVC-MR fluid cantilever structure is measured for the voltage range of a magnetic flux densities. The beam is initially deflected after packing the MR fluid in the cantilever structure due to the uniformly distributed mass of the MR fluid and the structure’s self-weight. The structure’s initial displacement is 5.59 mm when it is not loaded with external weights. An ultrasonic displacement sensor (HC-SR04) measures the displacement of the cantilever structure at its free end. At 0.026 T, 0.077 T, and 0.171 T, the initial displacement of the cantilever structure without external load is 4.43 mm, 2.78 mm, and 1.13 mm, respectively. The known weight (10 g, 20 g, 30 g, 40 g, and 50 g) is now placed at the structure’s free end. The displacement of each load at the free end is monitored using an ultrasonic displacement sensor. By subtracting the initial displacement from the sensor’s measured displacement value, the actual displacement is calculated. Figure 6 shows the experimental setup where ultrasonic displacement sensor is placed at bottom of the free end of the beam and the voltage of the electromagnet can be varied by using the variable transformer. For different magnetic field intensity, the corresponding deflection of beam with respect to the external load applied is measured.

## 6. Results and Discussion

### 6.1. Damping Properties

The damping properties such as damping ratio and logarithmic decrement were evaluated from the time domain plot of vibration response of different cantilever structure from the vibration response experiment. The time domain data acquired from accelerometer provides the amplitude of vibration. The Logarithmic decrement, δ is calculated by taking natural logarithm of the ratio of any two successive amplitudes. Since it is the free damped vibration response, the damping ratio, ε is calculated by $\varepsilon =\frac{\delta }{\sqrt{\left(4\pi^{2}+\delta^{2}\right)}}$. Table 2 represents the data recorded in different cantilever structure. It is found that damping ratio, logarithmic decrement and natural frequency are increased due to increase in magnetic flux density. The natural frequency of the cantilever structure is significantly shifted, which shows that magnetic field can be applied to shift the natural frequency away from the operating frequency of the structure to avoid resonance.

**Figure 7:**
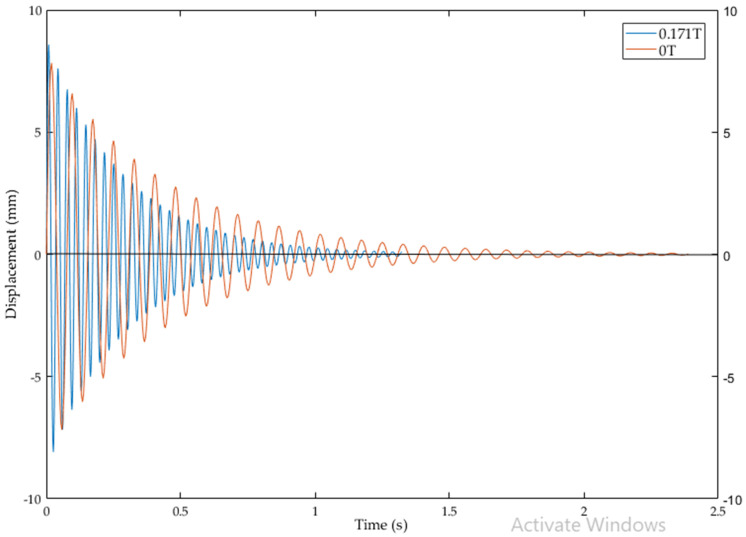
Vibration response of cantilever structure with MR fluid at 0 V (0 T) and 24 V (0.171 T).

The vibration response of cantilever structure with MR fluid at 0 T and 0.171 T is compared in Figure 7. It is proved that by increasing the magnetic field intensity the damping ratio and logarithmic decrement are increased. The damping ratio is increased by 44.5% due to the increase in magnetic field intensity from 0 T to 0.171 T. The vibration amplitude is suppressed with influence of external magnetic field. The plot shows that underdamping is achievable with MR fluid in these magnetic flux densities. Thus, these structures have applications limited to underdamping engineering problems.

### 6.2. Stiffness Variation

**Figure 8:**
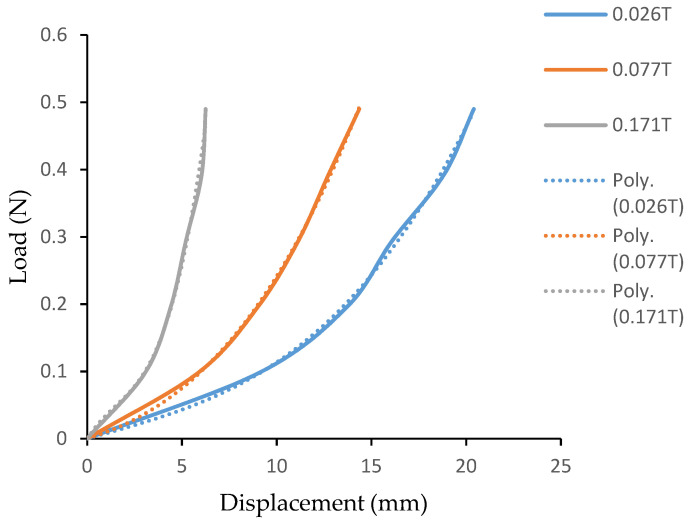
Load–displacement curve of cantilever structure with MR fluid for different magnetic field intensity.

The stiffness behavior of the structure with core layer as MR fluid at different magnetic field intensity is measured (Figure 8) from the stiffness characterization experiment. The deflection of flexible PVC-MR fluid beam for different external load with respect to applied magnetic field is studied. The load displacement curve shows non-linear elastic deformation. Due to its non-linear behavior, the load- displacement curve is constituted by a polynomial function using non-linear regression as shown in Table 3. The displacement decreasing for increase in magnetic flux density for same load applied is observed. It proves that the stiffness increases with increase in magnetic field intensity. This property allows the tunable stiffness nature of structure for the desired range. The stiffness characteristics of flexible cantilever structure shows that the use of magnetic responsive material as core is functional in control of damping and stiffness. The load–displacement curve is determined experimentally. The average stiffness is increased by 226.66% due to increase in magnetic field from 0 T to 0.171 T. Table 4 represents the field dependent stiffness of the cantilever structure with MR fluid. It is obtained by differentiating the polynomial function in Table 3. The notations y, x and k represent load, displacement and stiffness respectively. These polynomial functions will be used in development of controller for adaptive structures applications involving flexible PVC–MR fluid cantilever structure.

## 7. Conclusions

The flexible PVC-MR fluid cantilever structure with a stiffness tuned by external magnetic field is fabricated and experimentally validated. The structure is made up of flexible polyvinyl chloride as host structure with MR fluid as core. Vibration response of smart flexible structure is determined. The damping ratio increased by 44.5% due to increase in magnetic field intensity from 0 T to 0.171 T. The load–displacement curve is determined experimentally. The average stiffness is increased by 226.66% due to increase in magnetic field from 0 T to 0.171 T. The field dependent stiffness behavior is evaluated by curve fitting using nonlinear regression for load–displacement curve.

## Figures and Tables

**Table 1:** Properties of MR fluid.

Physical Properties	MR Fluid
Density (g/mL)	2.45
Viscosity (Pa·s)	0.29

**Table 2:** Vibration response of different cantilever structure.

Type of Cantilever Structure	Damping Ratio	Logarithmic Decrement	Natural Frequency (Hz)
With MR Fluid at 0 V (0 T)	0.0191	0.12	13.91
With MR Fluid at 24 V (0.171 T)	0.0276	0.174	29.24

**Table 3:** Load–displacement relationship approximated by polynomial function in the cantilever structure with MR fluid.

Magnetic Flux Density	Curve Fitting for Non-Linear Function	R^2^
0.026 T	y = 0.000042x^3^ − 0.000084x^2^ + 0.007975x + 0.000026	0.998282
0.077 T	y = 0.000054x^3^ + 0.001033x^2^ + 0.008433x + 0.000249	0.999571
0.171 T	y = 0.002705x^3^ − 0.012099x^2^ + 0.044597x − 0.000222	0.990199

**Table 4:** Stiffness approximated by polynomial function in the cantilever structure with MR fluid.

Magnetic Flux Density	Curve Fitting for Non-Linear Function $\left(k=\frac{dy}{dx}\right)$
0.026 T	k = 0.000126x^2^ − 0.000168x + 0.00795
0.077 T	k = 0.000162x^2^ + 0.0002066x + 0.008433
0.171 T	k = 0.008115x^2^ − 0.024198x + 0.044597

## Data Availability

The data presented in this study are available on request from the corresponding author. The data are not publicly available due to scholar pursuing doctoral program.
